# Targeted suppression of gibberellin biosynthetic genes *ZmGA20ox3* and *ZmGA20ox5* produces a short stature maize ideotype

**DOI:** 10.1111/pbi.13797

**Published:** 2022-03-09

**Authors:** Tomasz Paciorek, Brandi J. Chiapelli, Joan Yiqiong Wang, Marta Paciorek, Heping Yang, Anagha Sant, Dale L. Val, Jayanand Boddu, Kang Liu, Chiyu Gu, Lillian F. Brzostowski, Huai Wang, Edwards M. Allen, Charles R. Dietrich, Kelly M. Gillespie, Janice Edwards, Alexander Goldshmidt, Anil Neelam, Thomas L. Slewinski

**Affiliations:** ^1^ Bayer Crop Science Chesterfield MO USA; ^2^ Bayer Crop Science Woodland CA USA; ^3^ Present address: Department of Field Crops Science Institute of Plant Science Agricultural Research Organization The Volcani Center P.O. Box 15159 Rishon Lezion 7528809 Israel

**Keywords:** *GA20ox3*, *GA20ox5*, gibberellin, maize, miRNA, plant height, short stature, standability

## Abstract

Maize is one of the world’s most widely cultivated crops. As future demands for maize will continue to rise, fields will face ever more frequent and extreme weather patterns that directly affect crop productivity. Development of environmentally resilient crops with improved standability in the field, like wheat and rice, was enabled by shifting the architecture of plants to a short stature ideotype. However, such architectural change has not been implemented in maize due to the unique interactions between gibberellin (GA) and floral morphology which limited the use of the same type of mutations as in rice and wheat. Here, we report the development of a short stature maize ideotype in commercial hybrid germplasm, which was generated by targeted suppression of the biosynthetic pathway for GA. To accomplish this, we utilized a dominant, miRNA‐based construct expressed in a hemizygous state to selectively reduce expression of the *ZmGA20ox3* and *ZmGA20ox5* genes that control GA biosynthesis primarily in vegetative tissues. Suppression of both genes resulted in the reduction of GA levels leading to inhibition of cell elongation in internodal tissues, which reduced plant height. Expression of the miRNA did not alter GA levels in reproductive tissues, and thus, the reproductive potential of the plants remained unchanged. As a result, we developed a dominant, short‐stature maize ideotype that is conducive for the commercial production of hybrid maize. We expect that the new maize ideotype would enable more efficient and more sustainable maize farming for a growing world population.

## Introduction

The agricultural Green Revolution is considered one of the most pivotal points in the transformation of global agriculture, having a tremendous impact on worldwide food production, socio‐economic conditions, and environmental sustainability (Khush, 2001). This revolution was realized by the parallel implementation of advanced agronomic practices, modern fertilizers, and a new plant ideotype that dramatically improved lodging tolerance. In small grain crops like wheat and rice, increased inputs of fertilizers and increased planting densities promoted rapid stem elongation that made crops more prone to lodging, which caused significant economic losses associated with reduced yields, quality, and harvesting efficiency (Khan *et al*., 2019; Wang *et al*., 2012; Yang *et al*., 2009). To counter the unintended impacts of lodging, a reduced stature plant ideotype was conceptualized that dramatically improved overall plant sturdiness and enabled higher planting densities (Khush, 2001). Among the first crops that were developed to harness the power of these new architectural ideotypes were wheat (Peng *et al*., 1999) and rice (Monna *et al*., 2002; Sasaki *et al*., 2002; Spielmeyer *et al*., 2002). Since their introduction, both crops have been readily accepted by growers and have contributed to saving millions of hectares of land from being brought into agricultural production (Stevenson *et al*., 2013).

The molecular mechanisms underlying plant height reduction in wheat and rice involve biosynthesis and signal transduction of the endogenous plant hormone gibberellin (GA) (Hedden, 2003). Although GA regulates a vast array of developmental processes in plants (reviewed in Hedden and Thomas, 2016), its most apparent function is the control of stem elongation that determines plant height (Wang *et al*., 2017). GA was discovered in Japan as a fungal metabolite causing ‘foolish seedling disease’, which manifests as very rapid and excessive stem elongation in rice (Kurosawa, 1926; Yabuta and Sumiki, 1938). In the next decades, the underlying GA biosynthetic pathway was fully elucidated, and the functions of key genes were established (reviewed in Hedden, 2016).

In higher plants, early steps of GA biosynthesis are controlled by genes whose mutations often produce severely GA‐deficient plants with strong developmental and reproductive off‐types (Bensen *et al*., 1995; Koornneef and van der Veen, 1980; Winkler and Helentjaris, 1995). In later stages of GA biosynthesis, the activity of *gibberellin 20 oxidase* (*GA20ox*) genes regulates the production of bioactive GA (Coles *et al*., 1999; Huang *et al*., 1998). These genes are organized in multigene families with functionally redundant members that often show distinct expression patterns (Rebers *et al*., 1999; Rieu *et al*., 2008). Therefore, suppression of individual *GA20ox* genes may result in moderate GA deficiency making it a strategically attractive target to modify plant height in crops. The architecture of multiple agronomically important plant species like rice (Ashikari *et al*., 2002; Han *et al*., 2019), tomato (Xiao *et al*., 2006), apple (Bulley *et al*., 2005), potato (Carrera *et al*., 2000), and banana (Shao *et al*., 2019) has been improved (i.e., by dwarfing) through the modulation of the expression of *GA20ox* genes.

Maize (*Zea mays* L.) is critical for ensuring global food security because it provides, together with wheat and rice, at least 30% of the caloric requirements of more than 4.5 billion people worldwide (CIMMYT, 2011). Over the past decades, maize has seen significant improvements in production through hybridization, pathogen resistance, and higher planting density tolerance. However, high plant density increases intra‐plant competition for resources and reduces light availability to the lower canopy. This causes an increase in elongation and decrease in the diameter of internodes, making internodes slimmer and therefore more prone to lodging (Sher *et al*., 2018; Song *et al*., 2016; Xue *et al*., 2016). Stalk lodging in maize causes estimated yield losses ranging from 5% to 20% annually worldwide (Flint‐Flint‐Garcia *et al*., 2003). Therefore, reducing plant height in maize presents a large opportunity to improve crop standability to better withstand challenging weather and reduce crop loss. Short stature maize would also enable extended in‐season access for common, ground farming equipment to apply nutrients and crop protectants more precisely and more efficiently on an as‐needed basis. In turn, more strategic in‐season resource management would contribute to improved sustainability of maize production.

Although dwarfing alleles of genes in the GA pathway have been identified in maize, commercialization of such mutants has been challenging due to the extreme dwarf and pleiotropic effects of known mutant alleles (Bensen *et al*., 1995; Cassani *et al*., 2009; Chen *et al*., 2014; Fujioka *et al*., 1988; Wang *et al*., 2013). An additional obstacle in the commercialization of GA‐deficient maize mutants results from the GA‐dependent control of sex determination in maize flowers (Dellaporta and Calderon‐Urrea, 1994). Most reported GA‐deficient maize mutants to develop bisexual flowers (anthers in the ears and carpels in the tassels), which entirely abolish the yield potential of the crop (Bensen *et al*., 1995; Chen *et al*., 2014; Wang *et al*., 2013). In addition, most alleles identified for the key biosynthetic steps of the GA pathway are recessive in nature. This presents a challenge for the commercial production of a hybrid crop as these loci need to be fixed and introgressed into both male and female germplasm pools, which requires extensive resources. A dominant trait is desirable because it can be deployed on one side of the germplasm pool, making the launch of a new trait in commercial hybrids more cost and time effective. Therefore, dominant, finely tuned, and targeted suppression of GA biosynthesis in vegetative tissues is required to develop a short stature maize ideotype with the potential for commercialization.

Here, we report the development of short stature maize by transgenic manipulation of the expression of selected maize *ZmGA20ox* genes in commercial germplasm. We used a dominant, miRNA‐based suppression technology that allows for agronomic trait improvement when expressed in a hemizygous state, making it compatible for rapid introduction and field deployment in hybrid crops like maize. The suppression cassette selectively reduces expression levels of two target genes that underpin the biosynthesis of GA primarily in vegetative tissues such as stems and leaves. To further minimize impacts of GA reduction in reproductive organs, we expressed the suppression cassette under transcriptional control of the rice tungro bacilliform virus (RTBV) promoter, which showed the highest expression level within internodes in maize. Selective suppression of the target genes resulted in the reduction of GA levels and consequently led to the inhibition of cell elongation in internodal tissues of hybrid maize. As a result, we developed a maize ideotype with the desired reduction in stature. This maize crop system would enable more efficient farming and more sustainable crop management. Short stature maize has the potential to revolutionize the agricultural sector and improve food production for a growing world population.

## Results

### miRNA‐based suppression technology was used to target selected *ZmGA20ox* genes

**Figure 1:**
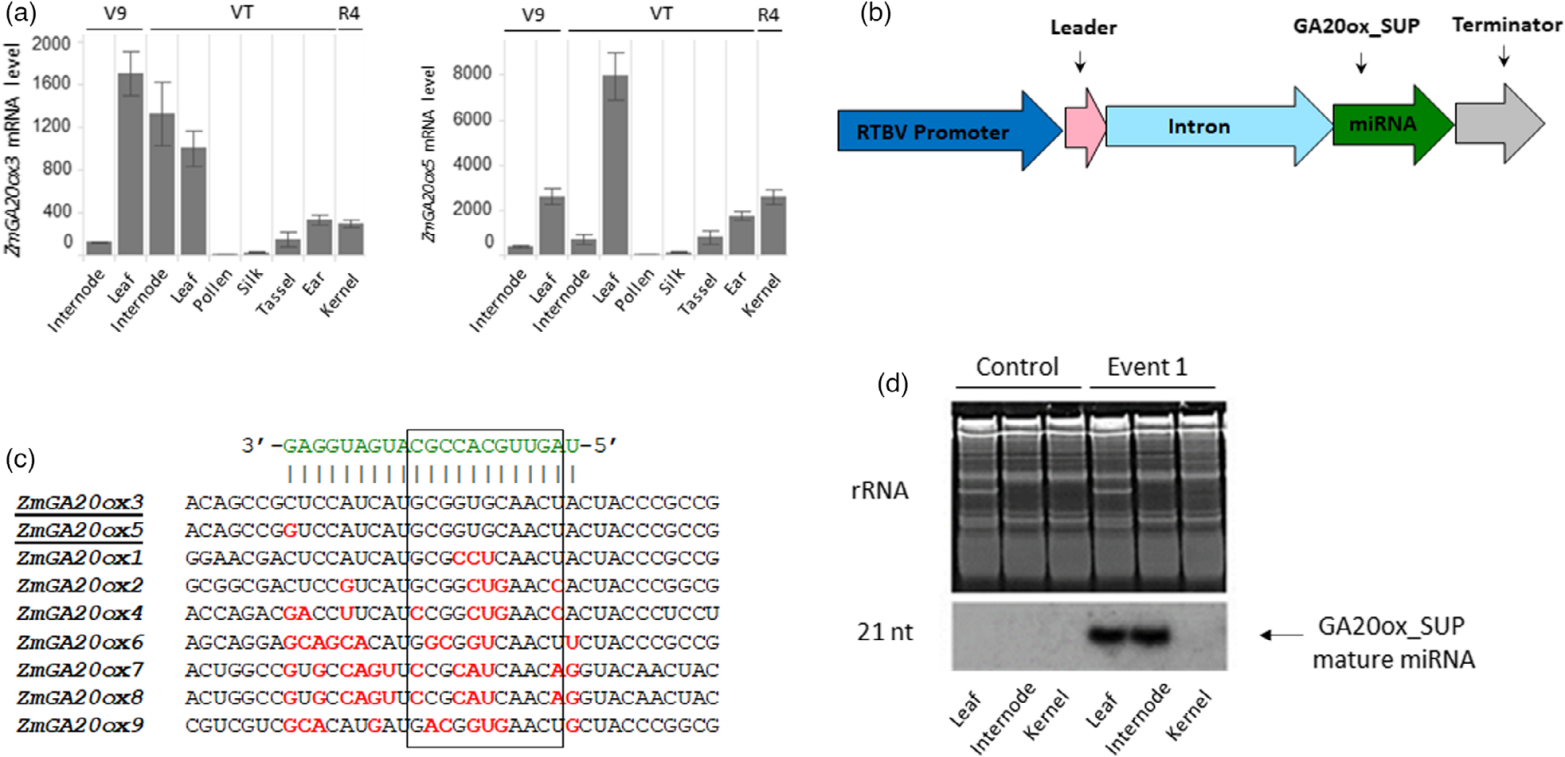
miRNA‐mediated suppression technology was used to develop short stature maize. (a) mRNA levels of *ZmGA20ox3* and *ZmGA20ox5* genes in different tissues of field‐grown maize plants determined with the qRT‐PCR assay. Error bars represent the standard error of the difference. V9, VT, and R4 refer to developmental stages. (b) Schematic illustration of the suppression cassette. (c) Nucleotide alignment of miRNA recognition site in nine *ZmGA20ox* genes identified in maize. The sequence in green indicates the GA20ox_SUP mature miRNA, the black outlined box marks the seed region of the miRNA recognition site, nucleotides in red represent mispaired nucleotides, and the underline marks two target *ZmGA20ox* genes. (d) Low molecular weight RNA blot detection of 21‐nucleotide mature miRNA from different tissues of tall control and transgenic event 1. rRNA represents ribosomal RNA control.

In maize, five major *ZmGA20ox* genes have been identified (Song *et al*., 2011); however, a search of the Maize Genetics and Genomics Database (https://www.maizegdb.org) suggested four additional putative *ZmGA20ox* genes (Portwood *et al*., 2019). The nine identified *ZmGA20ox* genes were further evaluated as potential targets for suppression. Based on the publicly available atlas of global transcription profiles for maize genes (Sekhon *et al*., 2011; Winter *et al*., 2007), we selected *ZmGA20ox3* and *ZmGA20ox5* which showed higher relative expression levels in vegetative tissues and lower levels in reproductive tissues (http.//bar.utoronto.ca/efp_maize/cgi‐bin/efpWeb.cgi). Higher vegetative expression of both genes was validated in Bayer’s maize germplasm (Figure 1a). In addition, both selected genes share high sequence homology with the rice *SD1* gene (*OsGA20ox2*), and all three genes group together in the same clade (Song *et al*., 2011). The rice *SD1* gene is considered the key semi‐dwarfing gene deployed in modern rice breeding (Monna *et al*., 2002; Sasaki *et al*., 2002; Spielmeyer *et al*., 2002). These factors identified *ZmGA20ox3* and *ZmGA20ox5* as the most likely candidates for gene suppression to reduce GA levels in maize stems without impacting reproductive tissues.

To reduce the transcript levels of both target genes, a miRNA‐based suppression technology was used. This approach enabled targeted suppression of two very close family members in a dominant manner while avoiding potential impact on other genes. A 21‐nucleotide microRNA termed ‘GA20ox_SUP’ (Figure 1b) with perfect complementarity to *ZmGA20ox3* and one mismatch with *ZmGA20ox5* (Figure 1c) was designed. This mismatch was mapped outside of the ‘seed‐region’ of the miRNA recognition site that is critical for the pairing of the miRNA and its target mRNA (boxed region on Figure 1c), and therefore comparable suppression efficiency was predicted for both target genes. No other members of the *ZmGA20ox* family were predicted to be suppressed by GA20ox_SUP (Figure 1c).

The GA20ox_SUP miRNA was expressed under transcriptional control of the RTBV promoter along with an intron sequence derived from the first intron of the maize gene encoding the heat shock protein 70 (Hsp70) used to enhance the efficiency of expression in plants (Figure 1b). Previous reports in rice showed transcriptional activity of this promoter was detected primarily in vascular bundles (Bhattacharyya‐Pakrasi *et al*., 1993; Yin and Beachy, 1995). This promoter retains its vascular bundle specificity in maize (Figure S1a), which results in a strongly enriched internode expression level due to a high number of bundles in that tissue (Figure S1b). In addition, the RTBV promoter has relatively low activity in reproductive tissues (Figure S1b), which makes it a more refined tool for targeted suppression of GA in vegetative tissues. Independent transgenic events containing the suppression cassette were generated through *Agrobacterium*‐mediated transformation based on the method described previously (Sidorov and Duncan, 2009). Representative, single copy insertion events developed in Bayer elite hybrid maize material with a hemizygous suppression cassette were used throughout this study. As expected, based on the RTBV expression pattern, mature GA20ox_SUP miRNA was readily detected by northern blot analysis in the internodes and leaf tissue but not in immature kernels (Figure 1d).

### GA20ox_SUP miRNA reduces the expression levels of *ZmGA20ox3* and *ZmGA20ox5*

**Figure 2:**
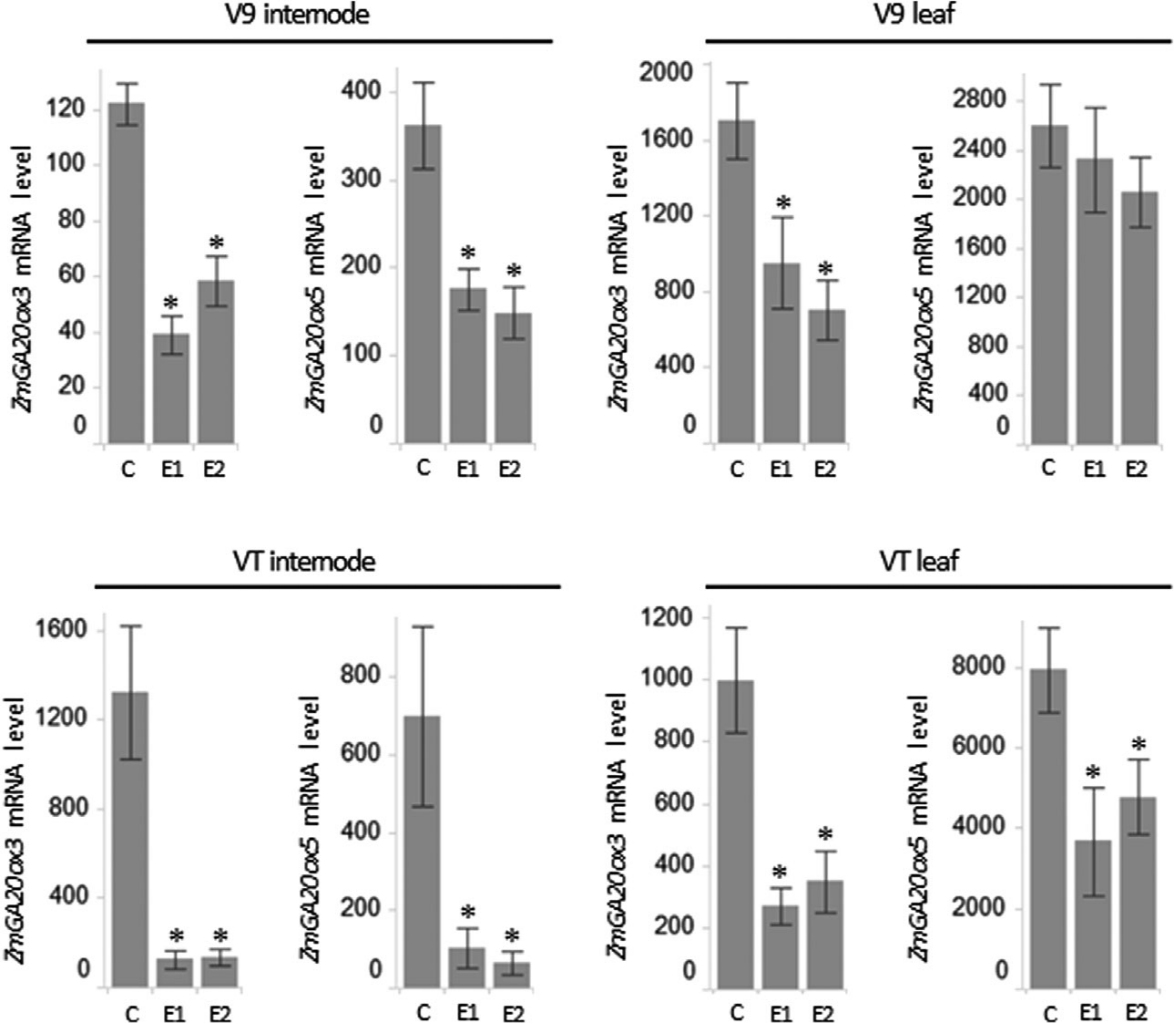
Quantitative expression levels for *ZmGA20ox3* and *ZmGA20ox5* in internodes and leaves collected from field‐grown transgenic events and tall control plants. Relative mRNA levels of the target genes were determined using a qRT‐PCR assay. Asterisks indicate the statistical significance between control plants and transgenic events at *P* < 0.05. V9 and VT refer to developmental stages. Error bars represent the standard error of the difference. C – tall control, E1 – event 1, E2 – event 2.

To examine the efficiency of GA20ox_SUP‐mediated down‐regulation of target genes, the expression levels of *ZmGA20ox3* and *ZmGA20ox5* were quantified using reverse transcription followed by quantitative real‐time polymerase chain reaction (qRT‐PCR). Expression was analyzed in a broad assortment of tissues that were collected from both the early (V9) growth stage, when active, GA‐dependent growth is dominant in vegetative maize tissues and from the later (VT) growth stage, when maize enters its reproductive phase. This analysis showed that the transcript levels of both target genes were reduced in most tested tissues (Figure 2 and Figure S2). In addition, to validate the predicted specificity of the GA20ox_SUP, the mRNA level of another member of the gene family – *ZmGA20ox1* – was quantified. This gene is predicted not to be suppressed by GA20ox_SUP and is the closest homolog to *ZmGA20ox3* and *ZmGA20ox5* in the maize genome. No significant impact on the expression of *ZmGA20ox1* was detected in most tissues tested (Figure S3). In some tissues, expression of *ZmGA20ox1* deviated from the control, which is most likely a result of the well‐documented compensatory mechanism of a GA‐dependent feedback loop that regulates the expression level of some GA 20‐oxidases (Desgagne‐Penix and Sponsel, 2008; Hedden and Kamiya, 1997; Itoh *et al*., 2002; Phillips *et al*., 1995; Xu, 1999).

### Suppression of *ZmGA20ox3* and *ZmGA20ox5* reduces the levels of bioactive GA

**Figure 3:**
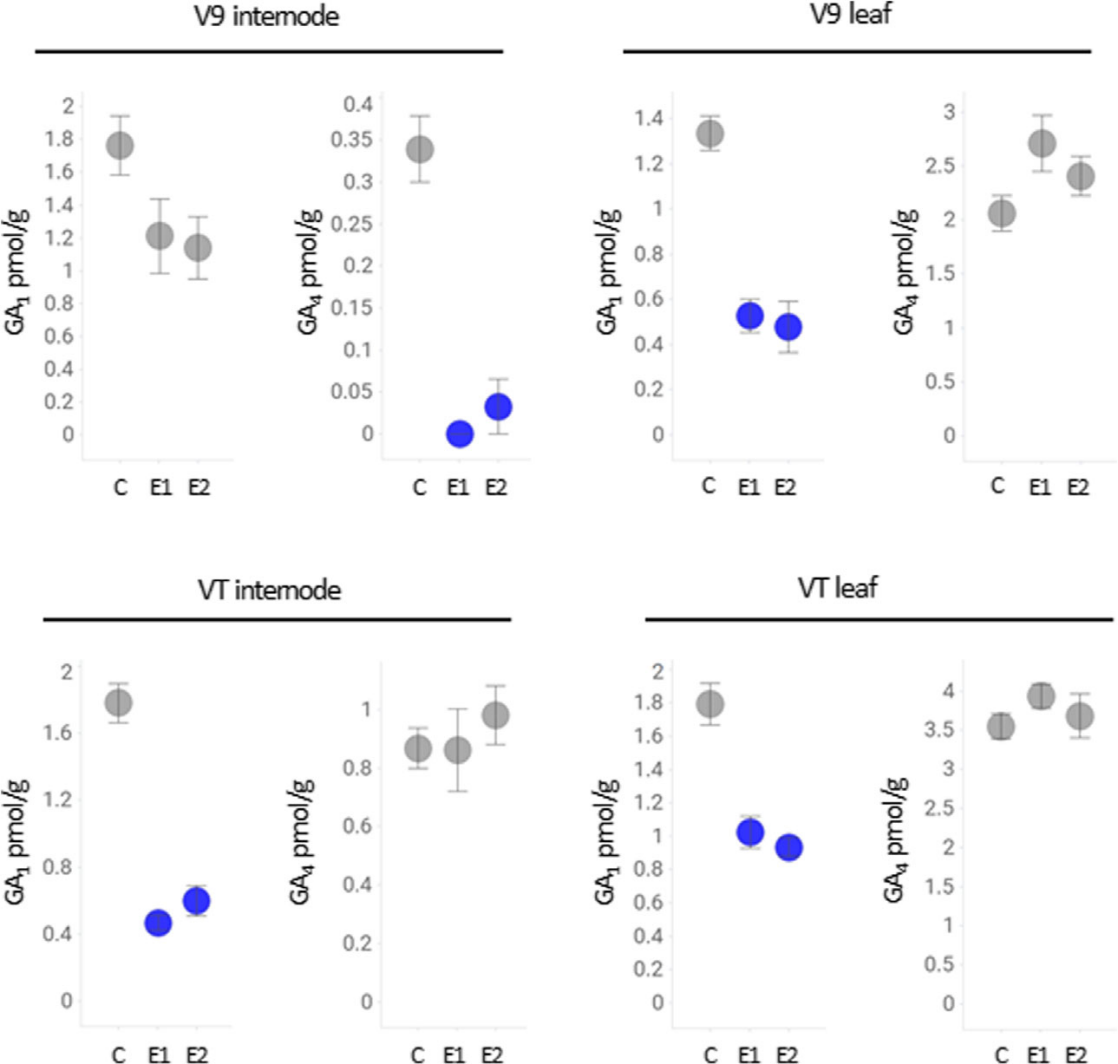
GA content in vegetative tissues collected from field‐grown transgenic events and tall control plants. LC–MS/MS analysis was used to quantify amounts of GA_1_ and GA_4_ in two independent transgenic events and control plants. Blue color indicates the statistical significance between control plants and transgenic events at *P* < 0.01, grey color indicates no statistical difference. V9 and VT refer to developmental stages. Error bars represent standard error. C – tall control, E1 – event 1, E2 – event 2.

To obtain a comprehensive view of the changes in GA levels in short stature maize, the levels of the major bioactive GAs were quantified in the same collection of vegetative and reproductive tissues that were used in the gene expression analysis. Expression of the suppression cassette impacted levels of GA_1_ and GA_4_ in vegetative tissues (Figure 3). In the early (V9) stage, GA_1_ was significantly reduced in leaves while GA_4_ was significantly reduced in internodes. At the VT stage, a decrease of only GA_1_ was observed in both internode and leaf tissue. Importantly, no significant reduction of GA levels was detected in reproductive tissues (Figure S4). These measurements demonstrate that the suppression of *ZmGA20ox3* and *ZmGA20ox5* expression results in the reduction of bioactive GA levels within the vegetative tissues of short stature maize.

### Reduction of GA levels changes the plant architecture resulting in short stature maize

**Figure 4:**
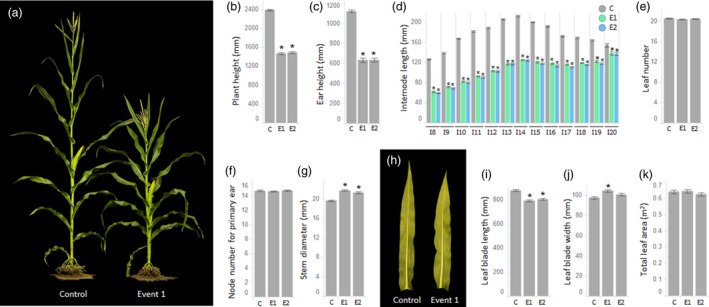
Phenotypic characterization of short stature maize grown in a test field at Jerseyville, Illinois (ILJE) USA. (a) Representative image of short stature maize and the tall control at R2 stage. (b) Plant height, (c) ear height, (d) individual internodes length, (e) leaf number, (f) node number for the primary ear, and (g) stem diameter measurements. (h) Representative image of leaf blades from a control plant and event 1. (i) Leaf blade length, (j) leaf blade width, and (k) total leaf area per plant measurements. Asterisks indicate statistical significance between control plants and transgenic events at *P* < 0.05. Error bars represent standard error. C – tall control, E1 – event 1, E2 – event 2, I8‐I20 on the graph (d) indicates numbering for consecutive internodes.

To determine the impact of GA reduction on maize architecture, detailed morphological measurements for selected agronomic traits were taken in open field conditions for both the tall control and short stature maize. To test the penetrance in different environments, measurements for a subset of traits were collected from two farm locations with different geographic characteristics: Jerseyville, Illinois, USA (ILJE, data presented in main figures) and Monmouth, Illinois, USA (ILMN, data presented in supplemental figures). These sites are in separate horticultural zones (https://planthardiness.ars.usda.gov/) that receive differential rainfall, experience differential temperatures, and are characterized by different soil profiles, that correlate with consistently different corn yield performance across both testing environments ( https://www.nass.usda.gov/Statistics_by_State/Illinois/Publications/Annual_Statistical_Bulletin/). Transgenic plants from both events showed an approximately 1/3 reduction in total plant height at each tested location, indicating consistent trait penetrance across the two environments (Figures 4a,b and S5a). The reduction of total plant height resulted from the cumulative shortening of all individual internodes both above and below the ear (Figure 4d). Importantly, as quantified by leaf number, no changes in internode number were observed in stems of the short stature maize (Figure 4e). Reduction of plant height caused a proportional reduction of ear height (Figures 4c and S5b) but did not change the primary ear node number (Figure 4f). Additionally, both events of short stature maize showed a statistically significant but minor increase in stem diameter (Figure 4g). Also, based on visual observations of field‐grown short stature maize, no excessive tiller formation was observed.

In addition to the internodes, a reduction of GA hormone was detected in leaves of short stature maize (Figure 3). To determine the impact of GA reduction on leaf morphology, the length and width of a single representative, the mid‐canopy leaf was measured on replicate plants grown in the field. GA deficiency in the leaves of short stature maize resulted in a reduction in length and a minor increase in the width of the leaf blade (Figure 4h–j). It is important to note that the observed dimensional changes in leaf shape did not significantly alter the total leaf area of short stature maize plants compared to controls (Figure 4k).

### Expression of GA20ox_SUP changes plant architecture without affecting the reproductive potential of short stature maize

To confirm that the expression of GA20ox_SUP miRNA did not impart any reproductive aberrations as reported in other severe GA mutants, both reproductive phenology and major yield parameters were collected in an open field setting. As evidenced by the number of days from planting to anthesis or silking, no agronomically significant changes were documented in the duration of time to these reproductive stages between the short stature maize and the tall control plants (Table 1a). Similarly, the critical parameters of ear morphology that determine maize productivity, such as ear area, ear diameter, ear length, kernel number, and single kernel weight, were found to be equivalent to those of tall control plants (Table 1b). Although some differences in ear components were detected, these differences were not consistent across both tested locations and therefore most likely represent environmentally driven variation. No bisexual female flowers were observed in any plants carrying the GA20ox_SUP cassette across all courses of testing (Figure S6). Importantly, in addition to lack of reproductive off‐types, seeds produced by short stature maize showed normal germination as demonstrated by equivalent seedling stand count in the field (Figure S7).

**Table 1:** Reproductive phenology and primary yield components of short stature maize grown in two field locations

Trait	Location	Event	Event mean	Control mean	*P*‐value
(a)					
Planting to 50% anthesis (days)	ILJE	E1	68.3	68.5	0.651
		E2	68.0	68.5	0.212
	ILMN	E1	66.7	66.5	0.679
		E2	65.8	66.5	0.512
Planting to 50% silking (days)	ILJE	E1	68.0	68.0	0.955
		E2	67.8	68.0	0.657
	ILMN	E1	66.0	66.9	0.292
		E2	64.5	66.9	0.048*
(b)					
Ear area (cm^2^)	ILJE	E1	83.9	81.0	0.045*
		E2	83.1	81.0	0.122
	ILMN	E1	87.5	86.8	0.720
		E2	83.7	86.8	0.195
Ear diameter (cm)	ILJE	E1	5.3	5.3	0.874
		E2	5.2	5.3	0.906
	ILMN	E1	5.2	5.1	0.478
		E2	5.1	5.1	0.579
Ear length (cm)	ILJE	E1	18.9	18.3	0.008*
		E2	18.7	18.3	0.080
	ILMN	E1	19.8	19.6	0.605
		E2	19.1	19.6	0.214
Kernels per ear (count)	ILJE	E1	587.4	602.2	0.572
		E2	624.2	602.2	0.435
	ILMN	E1	696.6	662.8	0.177
		E2	652.0	662.8	0.698
Single kernel weight (mg)	ILJE	E1	387.3	364.7	0.078
		E2	355.8	364.7	0.513
	ILMN	E1	329.2	345.2	0.013*
		E2	336.1	345.2	0.198

(a) The number of days to 50% anthesis and 50% silking and (b) selected ear component traits recorded for transgenic events and tall control plants. Asterisks indicate statistical significance between control plants and transgenic events at *P* < 0.05.

**Figure 5:**
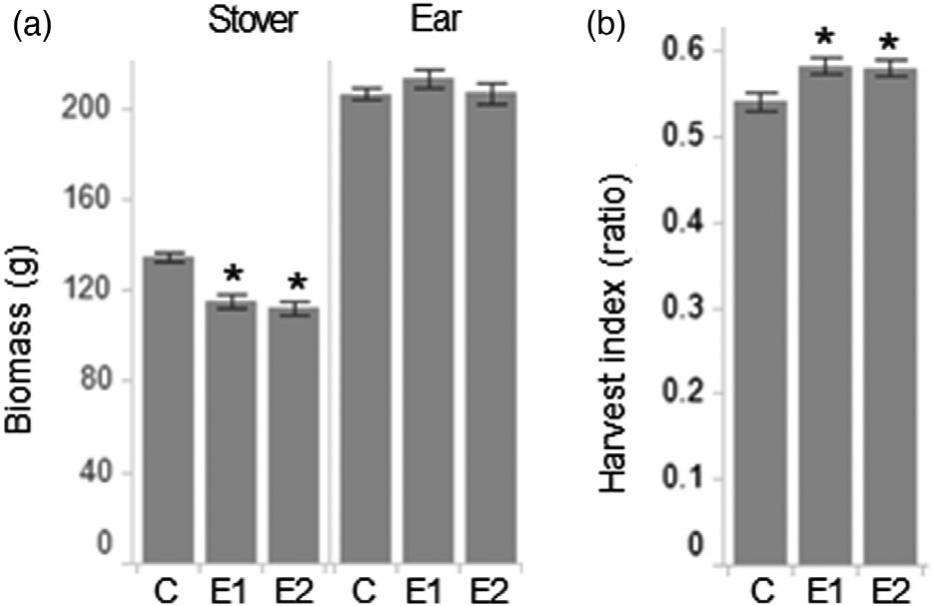
Biomass and harvest index of short stature maize grown in a test field at Jerseyville, Illinois (ILJE) USA. Dry biomass partitioning for stover and ear (a), and harvest index ratio (b) were measured for two transgenic events and tall control plants. Asterisks indicate statistical significance between control plants and transgenic events at *P* < 0.05. Error bars represent standard error. C – tall control, E1 – event 1, E2 – event 2.

At the whole plant level, the reduction of GA content in stem and leaves led to a decrease in plant dry stover biomass without affecting ear biomass (Figures 5a and S8a). The reduction in stover relative to ear biomass resulted in a consistent increase in harvest index in both events of short stature maize (Figures 5b and S8b). Taken together, morphometric measurements of field‐grown short stature maize did not show any unintended changes in key agronomic traits.

### Reduction of the GA levels inhibits elongation of internodal cells in short stature maize

**Figure 6:**
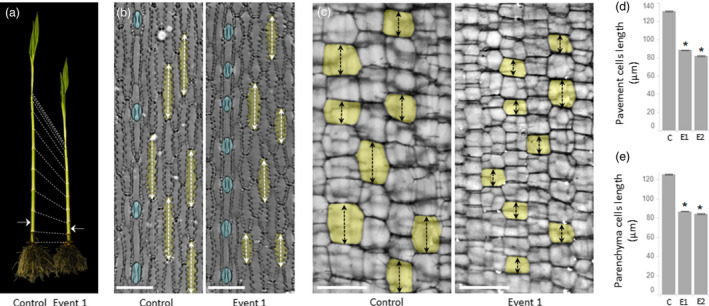
Cellular phenotypes in short stature maize internode. (a) Representative image of plants at V9 stage used for cell biology analysis. Dashed lines connect developmentally equivalent nodes; the arrows indicate internodes used for dissections. (b) Representative images of epidermal pavement cells and parenchyma cells (c) from 6th internode of control plants and event 1. Random pavement cells and parenchyma cells are pseudo‐colored yellow; stomata are pseudo‐colored blue. Dashed arrows exemplify longitudinal axes of measured cells. (d) Quantification of longitudinal axes of pavement cells and parenchyma cells (e) in control plants and two transgenic events. Asterisks indicate statistical significance between control plants and transgenic events at *P* < 0.05. Error bars represent standard error. C – tall control, E1 – event 1, E2 – event 2. The scale bar on (b) and (c) represents 100 and 250 µm, respectively.

To visualize the consequences of GA reduction at the cellular level, we quantified the longitudinal axes of epidermal pavement cells and parenchyma cells in the internodes of short stature maize. Both cell types have been previously shown to respond to changes in GA levels in monocots (Kaufman *et al*., 1969; Otani *et al*., 2013). The analysis showed a statistically significant reduction in the length of both pavement cells (Figure 6b,d) and parenchyma cells (Figure 6c,e) in both transgenic events. The data obtained from these two cell types indicates that the GA‐dependent reduction of cell length is likely the primary cellular mechanism that contributes to the shortening of individual internodes (Figure 4d) and, consequently, to reduction in plant height (Figure 4a,b).

### The reduced plant height of short stature maize can be rescued by exogenous GA application

To further confirm that the expression of GA20ox_SUP miRNA only impacts GA biosynthesis, a commercially available, bioactive GA was applied to tissues of developing short stature maize plants as has been demonstrated in the complementation experiments for other GA biosynthesis mutants (Ordonio *et al*., 2014; Wang *et al*., 2013). As shown in Figure S9, the reduced plant height phenotype was rescued in a dose‐dependent manner by exogenous application of GA. This indicates that the GA signalling pathway was not disrupted by the expression of the GA20ox_SUP miRNA.

## Discussion

The introduction of a reduced stature trait into essential food crops like wheat and rice resulted in the development of a new crop ideotype with improved standability in the field, which contributed to increases in crop productivity enabling farmers to produce more food on the same land (Hedden, 2003). In this study, we report the development of a new maize ideotype exhibiting characteristics of other reduced stature crops such as wheat and rice. We designed a miRNA to partially suppress the biosynthetic pathway for the plant hormone GA to enable commercial development of reduced stature hybrid maize without compromising its agronomic productivity. We accomplished this by combining several elements. First, from many genes involved in GA biosynthesis and signalling in maize, we focused on the *ZmGA20ox* family whose members have been successfully used to modulate the architecture of many agronomically relevant plants without compromising reproductive success. Second, from all putative members of the *ZmGA20ox* family, we selected two genes for suppression that are functional orthologs of the well‐documented, semi‐dwarfing rice *SD1* gene. Also, transcripts from these two target genes were abundant in maize stem and leaf tissues. Therefore, the primary function of these genes was presumed to be regulation of GA biosynthesis in these organs, which indicated their sizable contributions to stem elongation and plant height in maize. Third, to ensure high specificity of target suppression and limited ‘off‐target’ effects, we employed a miRNA‐based approach that was shown to suppress plant genes with very high precision (Carbonell *et al*., 2015; Schwab *et al*., 2006). This technology utilizes the same biogenesis and mechanism as endogenous miRNAs (Tiwari *et al*., 2014), and it has been successfully applied to effectively modulate agronomically important traits in monocotyledonous species (Warthmann *et al*., 2008). As anticipated, the activity of the suppression cassette reduced mRNA levels for both target genes in expected tissues. It is important to note that both the native expression of the *GA20ox* genes, as well as the activity of the RTBV promoter driving expression of the miRNA, which suppresses these genes, are dynamic and may have different relative quantities within the different tissues or time points that were sampled in the presented analyses. This could potentially affect the relative stoichiometry between the miRNA and target mRNA and, consequently, impact suppression efficiency in sampled tissues.

In higher plants, the final stages of the GA biosynthesis pathway branch into two parallel pathways that convert inactive GA precursors into the bioactive end‐products, GA_1_ and GA_4_ (Hedden, 2016). In most plant species, including maize, GA_1_ is the major bioactive form of GA controlling internode elongation (Phinney, 1985), and a reduction of the GA_1_ level in maize has been shown to reduce total plant height (Chen et al., 2019a, 2019b). In our study, we showed that miRNA‐based suppression of *ZmGA20ox3* and *ZmGA20ox5* primarily reduces GA_1_ levels in short stature maize tissues (Figure 3). The level of GA_4_, which in many species co‐occurs with GA_1_ (Sponsel and Hedden, 2010), was also reduced in V9 stage internode tissue, making the internode the organ with the most significant GA reduction. Consequently, a reduction in GA levels led to a decreased longitudinal axis of two types of internodal cells in short stature maize. This observation is consistent with cellular phenotypes described in dwarf GA mutants of lettuce (Waycott and Taiz, 1991), in two independent GA‐deficient rice mutants (Huang *et al*., 2010; Liu *et al*., 2018), a height‐reduced GA maize mutant (Chen *et al*., 2019), and in plants treated with chemical inhibitors of GA biosynthesis (Chen *et al*., 2019; Wang *et al*., 2014). Based on that cellular phenotype, we postulate that GA‐dependent suppression of cell elongation is the primary mechanism contributing to the shortening of individual internodes, thus producing short stature maize. Another change observed in the stem of short stature maize was an increase in internode diameter (Figure 4g). This trait has been shown to be positively correlated with reduced stalk lodging (Martin and Russell, 1984; Novacek *et al*., 2013). An increase in internode diameter could be a general consequence of reduced stature, as a similar phenotype was observed in other, semi‐dwarf maize mutant‐like *brachytic2* (Multani *et al*., 2003). That mutant showed no changes in GA level (Knöller *et al*., 2010) and instead was impacted in a different hormonal pathway (Multani *et al*., 2003). Importantly, no changes in internode number (Figure 4e) or primary ear node number (Figure 4f) were recorded in either event, which indicates no major alteration of developmental processes in stems of short stature maize.

Reduction of GA level in crops like wheat and rice can lead to an increase in tiller number which is an important agronomic trait for grain production in these crops (Appleford *et al*., 2007; Lo *et al*., 2008). Maize GA can also influence tiller production (Rood, 1985) and excessive tillering was reported in maize GA signalling mutant (Cassani *et al*., 2009). However, based on field observations, the reduced level of GA in short stature maize does not lead to a consistent change in tillering across testing experiments.

Decreased GA levels were also detected in the leaves of short stature maize. It has been shown that the GA pool in leaves plays a critical role in the determination of stem height, and its perturbation in leaves can directly affect stem elongation (Dayan *et al*., 2012). In the developing maize leaf, the abundance of GA forms a narrow peak at the boundary between division and elongation zones (Nelissen *et al*., 2012). Disturbance of this GA gradient can change the anisotropy of leaf growth, thus affecting directional leaf expansion (Sprangers *et al*., 2020). Monocot plants deficient in GA biosynthesis, such as maize *dwarf3* (Nelissen *et al*., 2012), liliaceous *Tricyrtis* sp. (Otani *et al*., 2013), and rice (Lo *et al*., 2017) were shown to produce shorter but wider leaf blades. In wheat, the semi‐dwarfing *Rht* genes encode components of GA signalling and an inverse linear relationship was demonstrated between the dosage of *Rht* alleles and the rate of leaf blade expansion (Keyes *et al*., 1989). Similarly, mutations in the maize GA signalling pathway also result in similar changes in leaf blade morphology (Cassani *et al*., 2009). Consistent with these previous observations, reduction in the GA levels detected in leaves of short stature maize also slightly altered leaf dimensions, however, those changes were not as dramatic as noted in severe GA mutations and did not affect total leaf area (Figure 4k).

One of the major roadblocks hindering the commercial success of GA‐deficient, reduced stature maize plants results from the simultaneous effects on vegetative and reproductive development by GA. Many previously identified maize mutants with perturbed GA biosynthesis or signalling show suppression of stamen abortion in the ear, resulting in the development of pistillate flowers eliminating the reproductive potential of the plant (Bensen *et al*., 1995; Cassani *et al*., 2009; Chen *et al*., 2014; Winkler and Freeling, 1994). With the partial and selective suppression of the GA biosynthesis pathway in short stature maize, we were able to overcome this obstacle as evidenced by the lack of GA changes in reproductive tissues (Figure S4), normal development of female flowers (Figure S6), and unaffected germination of harvested seeds (Figure S7), which resulted in grain production equivalent to tall control maize (Table 1b). This finding may be due to the presence of other *ZmGA20ox* genes that are differentially expressed in the reproductive tissues and possibly play a more dominant role in GA biosynthesis in those tissues and developmental stages.

Historically, despite great advances in breeding in recent decades, the harvest index in North American maize hybrids has seen only very modest increase (0.45–0.53) (Russell, 1991). Increases in yield potential have been largely the consequence of increased biomass production rather than increased harvest index (Hay, 1995). Suppression of GA production restricted to vegetative tissues of short stature maize led to the reduction of stover biomass without changes in grain weight, which resulted in an increase in harvest index of about 0.58–0.6, depending on location (Figures 5b and S8b). The increase in harvest index accomplished in short stature maize represents an improvement that could have an impact on commercial maize productivity.

Over the past decade, the demand for maize has grown tremendously (Shiferaw *et al*., 2011) and, with the continuous expansion of the human population along with the rapid growth for feedstocks, we are approaching the limits of predicted capacity for crop‐derived food production. It is estimated that by 2050, farmers will have to produce 50% more food than they produce today to accommodate increasing energy needs and the changing dietary habits of a growing population (FAO, 2017; Ray *et al*., 2013). This challenge will be amplified by quickly progressing changes in the global climate, which are expected to cause more frequent and more extreme weather events (Gastineau and Soden, 2009) that will directly impact crop productivity (Zhao *et al*., 2017). Therefore, the development of environmentally resilient crop technologies with improved management practices is now more critical than ever to ensure sustainable food production. The introduction of short stature maize into commercial maize production could play a pivotal role in increasing global maize production in a changing climate.

## Methods

### Construction of the expression cassette and generation of transgenic events

A miRNA‐based suppression cassette was generated according to published guidelines (Schwab *et al*., 2005). Expression patterns of *ZmGA20ox3* and *ZmGA20ox5* were validated in Bayer maize germplasm using MCS microarray and EST databases. Sequences of these two genes were aligned to other genes in the family and sequence regions unique to these two genes were identified as potential miRNA target regions so that a miRNA would not impact the expression of other *ZmGA20ox*. Based on these unique regions, GA20ox_SUP miRNA was designed using a customized miRNA design tool developed at Bayer. Calculated parameters like minimal free energy, GC%, and ΔΔG were used as selection criteria during the design of the miRNA (Allen *et al*., 2005). As a backbone for the expression of mature GA20ox_SUP miRNA, the sequence of miRMON13 (Heisel *et al*., 2008) was selected. Sequences of all components of the cassette are provided in Table S2.

### Field testing and trait analysis

Approval to conduct field studies in the United States was obtained from the United States Department of Agriculture. All field studies were performed in 2019 and 2020 in Jerseyville (ILJE) and Monmouth (ILMN), Illinois, USA. GPS co‐ordinates for respective locations were as follows: 2019‐ILJE (39.0614142, −90.3047379); 2019‐ILMN (40.8771441, −90.6218606), and for 2020‐ILJE (39.0673882, −90.3076824). Field studies did not involve or impact any endangered or protected species. F1 hybrid seeds used for all trials were generated in hybrid nurseries located in Kihei, Hawaii, USA. Hybrids were generated by crossing the female short stature maize events or tall female controls (having a genetic background identical to the short maize but not transformed with miRNA‐based suppression cassette) to a single male tester containing commercial insect protection traits. The same controls were used for molecular and phenotypic evaluations in this study. Planting dates were as follows: 2019‐ILJE (April 29), 2019‐ILMN (May 16), and 2020‐ILJE (May 3). The trials were designed as a randomized complete block design with 16 replications. However, to mitigate height differences between short stature maize events and the isogenic tall control plants, the material was blocked into separate, adjacently placed unreplicated blocks based on short or tall plant height status, with borders designed to prevent shading effects upon experimental plots. In 2019, experimental plots were one row wide by five feet long with a 30‐inch alley and 30‐inch row spacing. In 2020, experimental plots were three rows wide by five feet long with a 30‐inch alley and 30‐inch row spacing. All plots were seeded at a planting density of 36,000 plants per acre. Depending on the trait, 8–16 replications were measured; for the majority of traits, 8 plants per experimental plot were measured. Trial preparation and maintenance were designed to optimize grain yield based on standard agronomic practices for the region. Maintenance pesticides were applied as needed, and all maintenance operations were performed uniformly over the entire production area at a given field site. The complete list of all collected traits with a brief description of the methodology is available in Table S1.

Trait data were analyzed with the ASREML R package (VSN International Ltd, UK). Outliers were identified using the deleted studentized residual method, and outlier data points were discarded before further statistical analysis. Each data set consisted of multiple events and the corresponding wildtype control. Test and control entries were analyzed by trial location, using a linear mixed‐effects model. For model fitting, the transgenic event (or non‐transgenic wildtype as a special case) was considered a fixed effect, while replication and the residual (experimental error) were considered random effects. The statistical model used was:

Yir=μ+Ei+Br+εir,

where Yir = observation from the i‐th entry within the r‐th rep, µ = overall mean, Ei = effect of the i‐th entry, Br = random effect of the r‐th replicate, and εir = experimental error (residual) corresponding to the observation Yir, which is assumed to be independently distributed as a Normal Distribution with mean 0 and standard deviation σ.

Point estimates of the means of the transgenic event and wildtype control and *P*‐value for the hypothesis test of whether or not there is a statistically significant difference between transgenic versus wildtype control were provided as the inferences from the model.

### Tissue sampling in the field

Tissue samples for gene expression and hormone analysis were collected from the 2019 field trial located at Jerseyville (ILJE), Illinois, USA. Samples from three representative plants per each experimental plot were collected and pooled together with the exception of pollen for which five individual plants were pooled together; ten replications were collected. Immediately after collection, all tissues were placed in dewars containing liquid nitrogen; pollen samples were frozen on dry ice. After collection in the field, plant material was transported to a milling facility where each sample was milled on a grinder in chilled milling tubes with chilled steel balls. Afterward, milled samples were unloaded and cold‐dispensed into 96‐well plates for analysis. Samples were kept frozen throughout the process.

### Gene expression analysis by qRT‐PCR

Plant samples were collected at the stages indicated, frozen immediately in liquid nitrogen, and individually ground to a fine powder in liquid nitrogen. To extract total RNA from tissues, two types of isolation kits were utilized depending on tissue type. For leaf, internode, silk, tassel, and ear tissues, the Direct‐zol™ RNA MiniPrep Kit (Zymo Research, Irvine, CA) was used. For kernel and pollen tissues, the mirPremier microRNA Isolation Kit (Sigma–Aldrich, St. Louis, MO) was used. In all cases, RNA was extracted according to the manufacturer’s instructions for total RNA isolation. Extracted RNA was quantified using a Nanodrop spectrophotometer (Thermo Scientific, Wilmington, DE) and a subset of RNA samples from each tissue type was run on a 5300 Fragment Analyzer System (Agilent, Santa Clara, CA) to confirm that the RNA was of good quality with RIN values >7. RNA was converted to cDNA using the High‐Capacity cDNA Reverse Transcription Kit (Applied Biosystems™, Foster City, CA) with an anchored oligo dT primer according to the manufacturer’s instructions. To ensure that the reverse transcription kit was not saturated with the template, a representative subset of samples was measured to verify that the RNA concentration was between 10 and 200 ng/µL, and all samples were within this range. Two microlitres of RNA were added to a final reaction volume of 10 µL. Prepared cDNA was diluted threefold and used for expression analyses of genes by a TaqMan or an SYBR Green assay (see Table S3 for sequences of primers and gene IDs) using PerfeCTa® qPCR FastMix® II or PerfeCTa® SYBR® Green FastMix® Reaction Mixes (QuantaBio, Beverly, MA) according to manufacturer’s instructions. The thermal cycling conditions used for all probe‐based assays are 98 °C for 10 s; 40 cycles of 98 °C for 1 s, 60 °C for 5 s, and 72 °C for 30 s using a CFX Real‐Time Detection System (Bio‐Rad, Hercules, CA) and a melt curve was added to cycling conditions for the SYBR based detection of *ZmGA20ox5* to ensure a single amplicon was being detected. Each assay was validated using a standard curve of control cDNA to determine that amplification efficiency was 100 ± 5% and performed as expected. Specificity of the highly similar *ZmGA20ox3* and *ZmGA20ox5* genes was confirmed using cloned portions of each of these genes. The Ct value for each transcript was determined by regression using the Bio‐Rad CFX Manager software (Bio‐Rad, Hercules, CA). The relative quantities (RQ) of target genes were calculated by normalizing to the geometric mean of maize *elongation factor 1a* (*EF1a*, Lin *et al*., 2014) and maize *eukaryotic initiation factor 1A* (*eIF1A*) using the formula 2^‐(Target Gene Ct ‐ GeoMean Internal Normalizer Ct).

For each sample group and for each gene of interest (GOI), the normality assumption was checked by visual examination of residual plots and histograms. Levene’s test was applied to check the assumption of homogeneous variance. This was followed by a Studentized residual outlier removal method and results outside of −3.0 to 3.0 were excluded from the analysis. SAS (2012, software release 9.4 (TS1M1)) PROC MIXED was used for comparisons. Differences between sample groups were defined within the analysis of variance and tested using *t*‐tests at the 5% level of significance. The data are expressed as a mean of sample group relative quantities while the error bars shown are the calculated standard error of the difference.

### Endogenous GA level measurements

After plant tissues were milled, 0.3 g of each tissue sample was dispensed into a 1.8 mL glass tube under dry ice conditions. In a 96 well‐formatted rack, samples were then extracted with 1.25 mL of 1% ammonium hydroxide solution which included 2 GA internal standards (d2‐GA_1_ (1–1.5 pmol) and d2‐GA_4_ (1–1.5 pmol) purchased from OlChemim LTD, CZ), by incubating samples overnight (~16 h) under rotation mode at 4 °C. Following incubation, samples were centrifuged at 1000 rpm for 10 min at 4 °C. The resulting supernatants were loaded into a solid phase extraction cartridge 96‐well Oasis MAX plate (Waters, Milford, MA). Using a manifolder vacuum, the samples were passed through the MAX cartridge, and the eluent was discarded. The MAX cartridge wells were washed with 1 mL of 5% ammonium hydroxide followed by a 1 mL of 100% high‐performance liquid chromatography (HPLC) grade methanol wash under manifolder vacuum; the wash eluent was discarded. Fractions containing GA were collected from the MAX cartridge via elution with 3.2 mL of 2% formic acid in 100% HPLC grade acetone by gravity flow, and the remainder was captured by applying moderate negative pressure using a vacuum manifold. The eluent was dried under pure N_2_, resuspended in 200 µL of 0.1% formic acid (98% ACS grade) and 20% methanol in milli‐Q water, and filtered through a 96 well of 0.22 µm polyvinylidene fluoride filter plate (Millipore). The 96 deep well plate had 700 µL glass inserts in the well for collecting the filtered extract. Ten microlitres of each sample were injected into the ultra performance liquid chromatography (UPLC)/electrospray ionization‐mass spectrometry (ESI–MS)/MS (Sciex) for analysis. All GA hormones, and 2 internal standards (d2‐GA_1_ and d2‐GA_4_), were analyzed with a UPLC (Sciex‐Shimadzu) coupled with a Sciex 5500 Mass Spectrometer run in the Multiple Reaction Monitoring (MRM) method. An Acquity C‐18 BEH 100 × 2.1 mm, 1.7 µm (Waters) UPLC column was used with a 300 µL/min flow rate at 40 °C with the following gradient method applied: at 0–9 min (mobile phase B 20–100%), at 10 min (mobile phase B 100%), at 10.1 min (mobile phase B 20%), and at 12 min (mobile phase B 20%). Mobile phase A was 0.1% formic acid (98% ACS grade) in Milli‐Q water and mobile phase B was 0.1% formic acid (98% ACS grade) in HPLC grade methanol. The MS method was by ESI–MS/MS with ionization voltage negative −3000 V, source temperature 500 °C, and GA ions were monitored by a segmented MRM method. The parent ion mass for GA_1_ is 347.1, and the fragment ion mass is 273. For GA_4_, the parent ion mass is 331.05, and the fragment ion mass is 243. The analytical software for LC/MS is Analyst (Sciex). GA_1_ (OlChemim, CZ) and GA_4_ (Sigma–Aldrich) were used as standards for quantitation of the GA samples. The GA signal peak areas were carefully evaluated and integrated either automatically or manually based on mass, fragment mass, and retention time of the standard and internal standard. The final GA concentrations were calculated based on the calibration curves which ranged from 0.0001 to 0.029 pmol/µL for GA_1_ and GA_4_. Calculations were made with the SciexOS software (Sciex). Each GA’s calibration curve displayed a good linear fit, with *R* ^2^ linear regression values >0.99. Eight technical controls per 96 well plate for each hormone were also included and evaluated in the analytical process to ensure they meet the standard criterion of RSD < 10% in each case.

PROC MEANS in SAS (SAS 2012) was used to calculate the sample mean and standard error of control and transgenic event for each tissue type. Analysis of variance (ANOVA) was conducted according to the following model for each tissue type:

Yi=μ+Mi+εi.

where Yi = the hormone value of event i, µ = the overall mean, Mi = the effect of event i, and εi = the residual error. SAS PROC MIXED was used to fit the model separately for each tissue type. Pairwise comparisons between test event and control were defined within the ANOVA and tested using *t*‐tests.

### Extraction of total RNAs for northern hybridization

Plants were grown in a greenhouse with 50% relative humidity under 650–800 µE of light at 29 °C for 16 h and for 8 h of darkness at 25 °C. Plants were watered and fertilized as necessary. All collected plant samples were individually milled to fine powders under freezing conditions. Total RNA, including small RNAs, was extracted using TRIzol® Reagent (Invitrogen, Carlsbad, CA) according to the manufacturer’s instructions. RNA quality and quantity were assessed using a NanoDrop 1000 spectrophotometer (Thermo Fisher Scientific).

### Low molecular weight RNA blotting

Ten microgram of total RNA from each sample were mixed with the RNA loading Buffer (Thermo Fisher Scientific, Waltham, MA), which contained ethidium bromide (EtBr), was denatured at 95 °C for 5 min, cooled at 4 °C for 5 min, and loaded into the wells of a Criterion Precast 15% polyacrylamide TBE‐Urea gel (Bio‐Rad, Hercules, CA). The RNA molecules were separated using denaturing gel electrophoresis in a Criterion Cell (Bio‐Rad) with 1× TBE buffer and visualized using ultraviolet (UV) light. After gel photos were taken using a GelDoc‐It®e Imaging System (UVP LLC, Upland, CA), the RNA molecules were transferred onto positively charged nylon membranes via electroblotting in 1× TBE buffer with a Criterion Blotter (Bio‐Rad) according to the manufacturer’s instructions. Two digoxigenin (DIG)‐labelled DNA probes were synthesized from the miRNA gene using a PCR DIG Probe Synthesis Kit (Roche, Basel, Switzerland) with two pairs of gene‐specific primers (GGCAGAGCCGTGCCCGTCTCAT and ATAAGCAATACATTTCTCCATCATGCGGTGCAAC, ATTCAGCAATATAATGTTCCTCCATCATCCAGTGCAATTA and TTCTTCACATCCTATAGAGAAACAAGCAAACAAAATGTTTTCTCTAG, respectively). The two 148 or 592 bp long probes, which cover the entire GA20ox_SUP primary miRNA sequence including the 3′UTR, were purified using the PureLinkTM Quick Gel Extraction and PCR Purification Combo Kit (Thermo Fisher Scientific). Purified DIG‐labelled DNA probe concentrations were measured using a NanoDrop spectrophotometer (Thermo Fisher Scientific) and probes were stored at −20 °C until use. Pre‐hybridizations were conducted at 42 °C with rotation in DIG Easy Hyb (Sigma–Aldrich) for 2 h. The DIG‐labelled DNA probes were denatured at 98 °C for 5 min, cooled at 4 °C for 5 min, and then added to the hybridization buffer at a final concentration of 20 ng/mL. Hybridizations were then carried out at 42 °C overnight with rotation in a DIG Easy Hyb (Sigma–Aldrich). Post‐hybridization washes were conducted at 50 °C with the DIG wash and block buffer set (Sigma–Aldrich), and DIG detection was performed using reagents and Lumi‐Film X‐ray films (Roche).

### Microscopic assessment of maize stem cells

Microscopic analysis of cell dimensions was conducted on V9 growth stage plants collected from the 2019 field trial located at Jerseyville (ILJE), Illinois, USA. From early stages, the developmental progression of all plants was tracked to ensure that developmentally corresponding internodes were selected for microscopy analysis. For parenchyma cells analysis, longitudinal hand‐sections were obtained from fresh tissue at the middle of internode #6 using a razor blade. Tissues were incubated in 0.05% Toluidine Blue for 1 min, washed three times in water for 5 min each, and were placed on microscopic slides for assessment. The region for imaging was selected based on the distance between the center and edge of the tissue. At least 5–8 images were taken from selected regions. Bright‐field images were taken with a 40× objective on a Leica M205 stereomicroscope (Leica Microsystems, Wetzlar, Germany). Cell length was measured using ImageJ (Schneider *et al*., 2012), by marking cell walls and measuring the longitudinal distance between them. All cells with clearly visible cells walls were measured, excluding vascular bundles and cells directly adjacent to vascular bundles. Material for epidermal cell analysis was prepared by taking 2 cm long epidermal impressions from the middle of the stem using clear nail polish and transparent tape according to Hoogendoorn *et al*., 1990. Impressions were mounted on microscopic slides and imaged with a 20× objective on an EVOS XL bright field microscope (EVOS XL core imaging system, Thermo Fisher Scientific). Cell measurements were taken using ImageJ (Schneider *et al*., 2012), by marking cell edges and measuring the distance between them. Across the entire peel, all cells with clearly visible cell edges were measured, excluding stomata cells.

### Exogenous GA application

Seeds were sown in 96 well planting trays, transplanted into 2‐gallon pots at 14 days after germination, and grown in a greenhouse with high‐pressure sodium lamps for supplemental lighting. Lights were set to deliver a 16 h photoperiod starting 16 h before dusk and turning off during daylight hours when the natural light intensity was above 400 Watt/m^2^. Temperature setpoints were 30 °C and 20 °C for day/night, respectively. Plants were arranged in a block of 10 north‐south rows of 21 ‘experimental’ plants flanked on the south end of each row with 2 untreated ‘border’ plants. In addition, this block was flanked on the east and west sides by a single row of 23 identically spaced untreated ‘border’ plants. The border plants were employed to minimize edge‐effects on plant height; these were additional plants from one of the transgenic events in the same hybrid background and were chosen because they would be of similar height as the ‘experimental’ plants. To minimize within‐replicate environmental variation, the 10 north‐south rows of experimental plants were treated as separate replicates in the analysis because the primary thermal gradient in the greenhouse was in the east‐west plane. The north‐south position of each of the entries within the 10 rows/replicates of 21 ‘experimental’ plants was randomized for each replicate, as was the order of the 3 treatments in the blocks of 3 plants (0, 250, or 1000 ppm gibberellic acid; GA_3_). Stock GA_3_ application solutions were prepared containing either 250 or 1000 ppm GA_3_ in the form of RyzUp SmartGrass (Valent Biosciences, Libertyville, IL), which was dissolved in deionized water and 0.05% crop oil concentrate (Loveland Products, Loveland, CO) adjuvant. All ‘experimental’ plants to be sprayed with the same rate of GA_3_ (250 or 1000 ppm) were removed from the randomized array described above and sprayed in sets of up to 14 plants in a 1.5 m × 2.5 m spray zone. 210 mL of the stock application spray solution was applied evenly across the spray zone containing the plants to be sprayed. Uniform application was made at 35 PSI using a paint‐ball back‐pack CO_2_ powered sprayer (PB201; R&D Sprayers, Bellspray, Inc., Opelousas, LA) equipped with 4 nozzle boom at 38 cm nozzle spacing employing Teejet 8002VS spray nozzles. All plants were sprayed on the same day when the majority of plants were at the V8 growth stage. Plants were returned to the randomized array after the spray applications had dried.

### Analysis of RTBVpro‐GUS reporter line

To investigate the expression pattern of the RTBV promoter in maize, the RTBV promoter sequence was transcriptionally fused with the *uidA* gene (Novel and Novel, 1973) encoding β‐glucuronidase (GUS) according to Jefferson, *et al*., 1987. The *uidA* coding sequence was optimized for expression in plant cells and harbors a processable intron derived from the potato light‐inducible tissue‐specific ST‐LS1 gene (Genbank Accession: X04753). Two hybrid events carrying the RTBVpro‐GUS reporter construct were generated in the same manner as short stature maize events. Both events were analyzed, and data from one event is reported in the publication. To quantitatively assay GUS enzymatic activity, approximately 50 mg fresh weight of tissue was lyophilized and powdered. Total protein was extracted from the powdered tissue using 500–800 µL of 100 mm KPO_4_ (pH 7.4) extraction buffer (1 mm NaEDTA, 0.1% lauryl sarcosine, 0.1% Triton 100×, 0.05% glycerol, 2% PVP, 10 mm β‐mercaptoethanol, and 0.2 mm PMSF). The total protein concentration was determined using a Bradford protein assay according to the manufacturer’s instructions (Bio‐Rad). To assay for GUS activity, 1–3 µg of the total protein extract was incubated with 50 nmol 4‐methylumbelliferyl‐β‐d‐glucopyranosiduronic acid (MUG) substrate (Sigma–Aldrich) in a 50 µL reaction at 37 °C for 1 h. The reaction was stopped by the addition of 350 µL 0.2 m sodium carbonate. The fluorescent product was measured with excitation at 365 nm and emission at 445 nm using a FLUOstar Omega microplate reader (BMG Labtech, Offenburg, Germany). The resulting values were converted to pmol of the fluorochrome 4‐methyl umbelliferone (4 MU) with a standard curve and normalized to total protein. GUS enzyme activity is reported as pmol 4 MU/µg total protein/h.

To visualize RTBVpro‐GUS reporter expression in maize tissues, selected organs were collected and sectioned. Tissue sections from leaves (90–120 μm thickness) were produced using a sliding microtome (Leica); tissue sections from internodes (0.5–1 mm thickness) were produced with a razor blade. Sections were submerged in GUS staining solution: (1 mg/mL X‐Gluc (5‐bromo‐4‐chloro‐3‐indolyl‐b‐glucuronide) (Sigma–Aldrich), 25 μm potassium ferricyanide, 2.5 μm potassium ferrocyanide, 0.05% Triton X‐100 (v/v) in 50 mm potassium phosphate buffer (pH 7.4)). The tissues were incubated in the staining solution at 37 °C for 5 h. De‐staining was performed by incubating tissues in 70% EtOH: glacial acetic acid (1:1 v/v) overnight followed by 70% EtOH. The tissues were imaged under a stereo dissecting microscope (Nikon) and a compound microscope (Nikon).

## Conflict of interest

This work was supported by Bayer Crop Science and was conducted in collaboration with BASF. Both companies provided financial, logistical, and regulatory support. All authors are or were affiliated with Bayer or Monsanto Company.

## Author contributions

E.M.A. and H.W. conceived the project concept, T.P. and T.L.S. designed experiments and conducted scientific analysis, C.R.D., K.M.G., and J.E. coordinated the project, B.J.C., J.Y.W., M.P., H.Y., A.S., D.V., J.B., A.G., and A.N. performed the experiments, T.P., B.J.C., J.Y.W., M.P., H.Y., A.S., D.V., K.L., C.G., and L.F.B. analyzed the data, T.P., and T.L.S. wrote the manuscript with input from other authors. All authors read, revised, and approved the final manuscript.

## Supporting information


**Figure S1** Expression pattern of RTBVpro‐GUS reporter in tall hybrid maize.

**Figure S2** Quantitative expression levels for *ZmGA20ox3* and *ZmGA20ox5* in reproductive tissues collected from field‐grown transgenic events and tall control plants.

**Figure S3** Quantitative expression level for *ZmGA20ox1* in vegetative and reproductive tissues collected from field‐grown transgenic events and tall control plants.

**Figure S4** GA content in reproductive tissues collected from field‐grown transgenic events and tall control plants.

**Figure S5** Phenotypic characterization of short stature maize at R2 stage grown in a test field at Monmouth, Illinois (ILMN), USA.

**Figure S6** Morphology of R6 ears collected from field grown tall control and two transgenic events of short stature maize demonstrating lack of bisexual female flowers.

**Figure S7** Seedling stand count measured at V3 stage for short stature maize and tall control plants are grown in two field locations.

**Figure S8** Biomass and harvest index collected from transgenic events and tall control plants grown in a test field at Monmouth, Illinois (ILMN), USA.

**Figure S9** Complementation of short stature phenotype with the application of exogenous GA.

**Table S1** List of all traits collected during phenotypic characterization of short stature maize in field conditions.

**Table S2**. List of sequences used to develop the miRNA‐based GA20ox_SUP suppression cassette.

**Table S3**. List of primers and gene IDs used for qRT‐PCR analysis.
