# Supplementary figures and images for: Targeted suppression of gibberellin biosynthetic genes ZmGA20ox3 and ZmGA20ox5 produces a short stature maize ideotype
Source: Plant Biotechnol J. 2022 Mar 9;20(6):1140–53. doi: 10.1111/pbi.13797 (PMC9129074; doi:10.1111/pbi.13797)

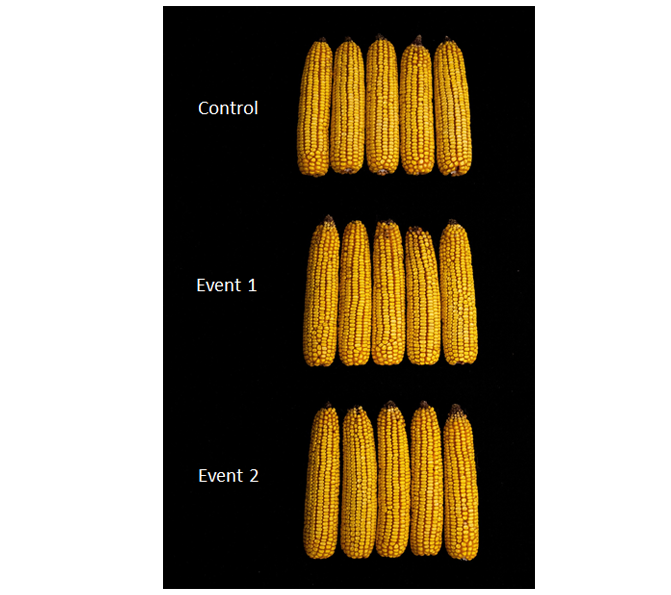

Supplement: Supplementary file 6 — Figure S6 Morphology of R6 ears collected from field grown tall control and two transgenic events of short stature maize demonstrating lack of bisexual female flowers. [file PBI-20-1140-s002.tif]

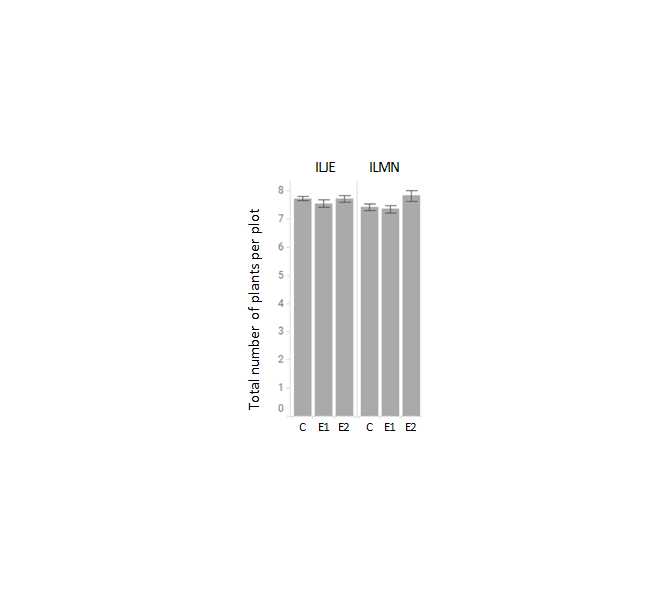

Supplement: Supplementary file 7 — Figure S7 Seedling stand count measured at V3 stage for short stature maize and tall control plants are grown in two field locations. [file PBI-20-1140-s008.tif]

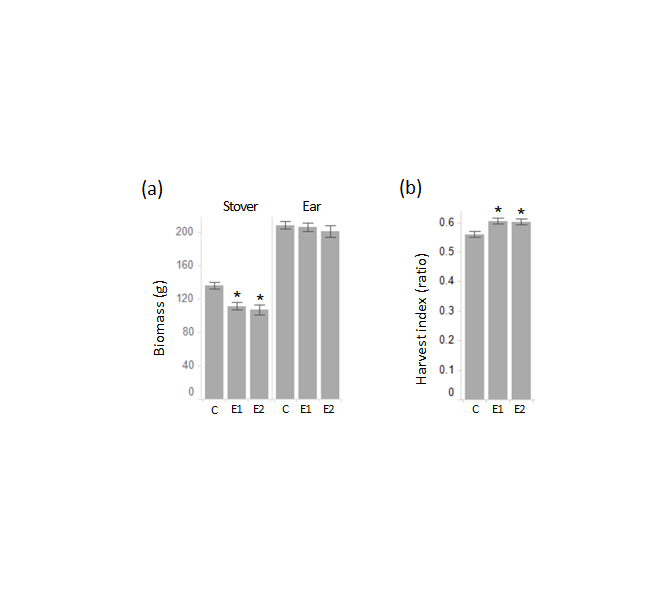

Supplement: Supplementary file 8 — Figure S8 Biomass and harvest index collected from transgenic events and tall control plants grown in a test field at Monmouth, Illinois (ILMN), USA. [file PBI-20-1140-s001.tif]

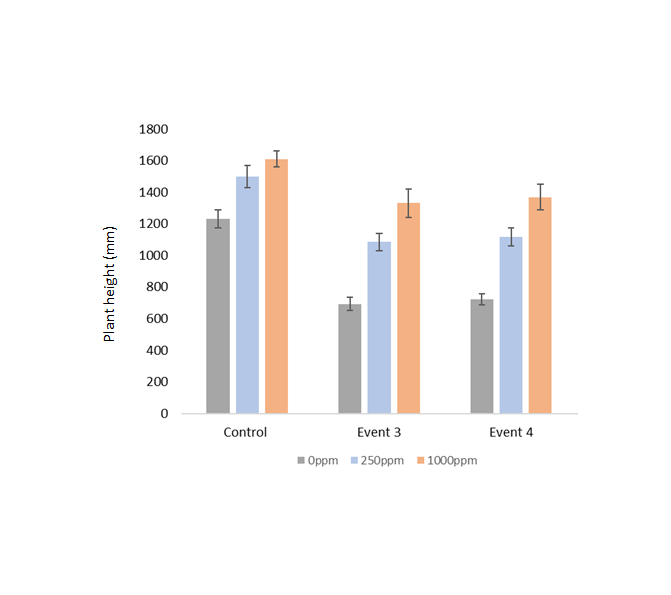

Supplement: Supplementary file 9 — Figure S9 Complementation of short stature phenotype with the application of exogenous GA. [file PBI-20-1140-s009.tif]
